# A Single-Center Pilot Study to Classify Signs of Dorsal Hand Aging Using 3 Grading Scales

**DOI:** 10.1093/asjof/ojab059

**Published:** 2022-02-17

**Authors:** Christine E Wamsley, Nicole Vingan, Jennifer Barillas, Abby Culver, David M Turer, Jeffrey M Kenkel

**Affiliations:** Department of Plastic Surgery, UT Southwestern Medical Center, Dallas, TX, USA; Department of Plastic Surgery, UT Southwestern Medical Center, Dallas, TX, USA; Department of Plastic Surgery, UT Southwestern Medical Center, Dallas, TX, USA; Department of Plastic Surgery, UT Southwestern Medical Center, Dallas, TX, USA; Department of Plastic Surgery, UT Southwestern Medical Center, Dallas, TX, USA; Department of Plastic Surgery, UT Southwestern Medical Center, Dallas, TX, USA

## Abstract

**Background:**

While validated scales must be created in order to systemically evaluate patients and quantify outcomes of aesthetic hand treatments, scales currently available are limited to the analysis of volume loss alone.

**Objectives:**

The purpose of this study was to develop 3 validated scales for the assessment of dorsal hand aging that also take into consideration wrinkling and pigmentation.

**Methods:**

Fifty (50) healthy volunteers (40 females and 10 males) with Fitzpatrick skin types I-IV were recruited, and standard photographs of their left and right dorsal hands were taken with a Nikon D7100 (Nikon; Minato, Tokyo, Japan) camera. Using 25 randomized photographs, 11 plastic surgery physicians (3 chief residents, 6 senior residents, and 2 aesthetic surgery fellows) were trained on the 3 scales under investigation as well as the already-validated Merz Hand Grading Scale (MHGS). The evaluators then viewed the remaining 75 photographs independently and assigned a grade for each of the 4 scales to each photograph. Inter-rater variability was calculated for each scale.

**Results:**

The Kappa score for the MHGS was 0.25, indicating fair agreement; 0.40 for wrinkle scale, indicating fair agreement; and 0.48 and 0.46 for the pigmentation density and intensity scales, respectively, indicating moderate agreement (*P* < 0.001).

**Conclusions:**

The results show that after receiving training, the inter-rater agreement for the 3 scales under investigation was similar or slightly higher than that for the MHGS. These 3 photographic classification systems can be used consistently and reliably to characterize multiple signs of dorsal hand aging.

**Level of Evidence: 2:**

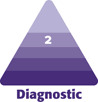

Along with the face, neck, and chest, the dorsal hands are also visible to signs of aging.^1,2^ As in other parts of the body, aging of the dorsal hand is characterized by gradual loss and disorganization of collagen, elastin, and other connective tissues.^3-6^ Decreased dermal collagen results in skin laxity and the appearance of crepiness and wrinkles.^3,5^ Additionally, photodamage, most due to ultraviolet radiation, causes degradation of elastic fibers and dark spots.^5,7^ Finally, soft tissue atrophy and resultant volume loss expose underlying structures, such as veins, tendons, and bony prominences.^6-9^

A number of hand rejuvenation procedures are available to combat the aging process by addressing 3 overarching concerns—epidermal tone, dermal collagen levels, and soft tissue volume.^5^ Topical agents, such as hydroquinone, tretinoin, vitamin C, and alpha-hydroxy acid, and abrasive methods, including chemical peels^10,11^ and dermabrasion,^12^ have been shown to be effective for epidermal rejuvenation and instigating cell turnover.^5,7^ Lasers and other light-based treatments can also address surface changes and signs of photoaging.^5,7,13^

Injectable dermal fillers and autologous fat grafting may add volume to soften the outline of deeper structures.^5,7,13,14^ Finally, sclerotherapy or endovenous vascular ablation can be performed to remove persistent or prominent veins.^5,8^

In order to systemically evaluate patients and quantify outcomes of aesthetic hand treatments, validated scales specific to the area of concern must be developed.^1^ In reviewing the literature, there are 3 scales currently validated for the assessment of dorsal hand aging—the Merz Hand Grading Scale (MHGS),^1,3^ the Allergan Hand Volume Deficit Scale,^6^ and the Hand Volume Rating Scale (HVRS).^9^ As the names suggest, all 3 scales are limited to evaluating the volume loss. As other factors also contribute to the appearance of aged hands, such as wrinkling, textural roughness, and general dyschromia, more scales that take these additional components into account must be created. These scales can be used in combination to more precisely quantify the different effects of dorsal hand treatments.

The objective of this study is to develop 3 validated scales for the assessment of signs of dorsal hand aging. The 3 scales under investigation are the Dorsal Hand Wrinkling Scale, the Dorsal Hand Pigmentation Density Scale, and the Dorsal Hand Pigmentation Intensity Scale. The goal is to determine whether photographic classification systems of wrinkles and spots, both density and intensity, can be used consistently to characterize the dorsal hand.

## METHODS

### Study Design

The single-center, pilot study protocol was approved by the Institutional Review Board at the University of Texas Southwestern Medical Center and conducted between September 2020 and January 2021; in October and November 2021, the study was expanded to include more photograph evaluators and increase statistical power. Fifty (50) patients (40 females and 10 males) above the age of 18 with Fitzpatrick skin types I-IV and with evaluable left and right hands were recruited from the Department of Plastic Surgery clinic. Exclusionary criteria included any unique features on the dorsal hands, such as scars, tattoos, or excessive hair, that could potentially identify the patient or interfere with classification. As no other demographic information was collected, the Institutional Review Board deemed verbal consent permissible. Therefore, verbal consent was provided, by which the patients whose photographs were used in scale evaluations agreed to the use and analysis of their data. Additionally, written consent was provided by the individuals whose hands were used to create the scales under investigation for the purposes of this publication. After consent was obtained, patients were taken to the Department of Plastic Surgery professional photography where separate photographs of their dorsal left and right hands were taken using a Nikon D7100 (Nikon; Minato, Tokyo, Japan). To maintain uniformity, patients were asked to remove any jewelry on their wrists or fingers and instructed to lay their hands on a blue board. Photographs were numbered sequentially in the order they were taken and remained anonymous.

### Scale Development

**Figure 1.**
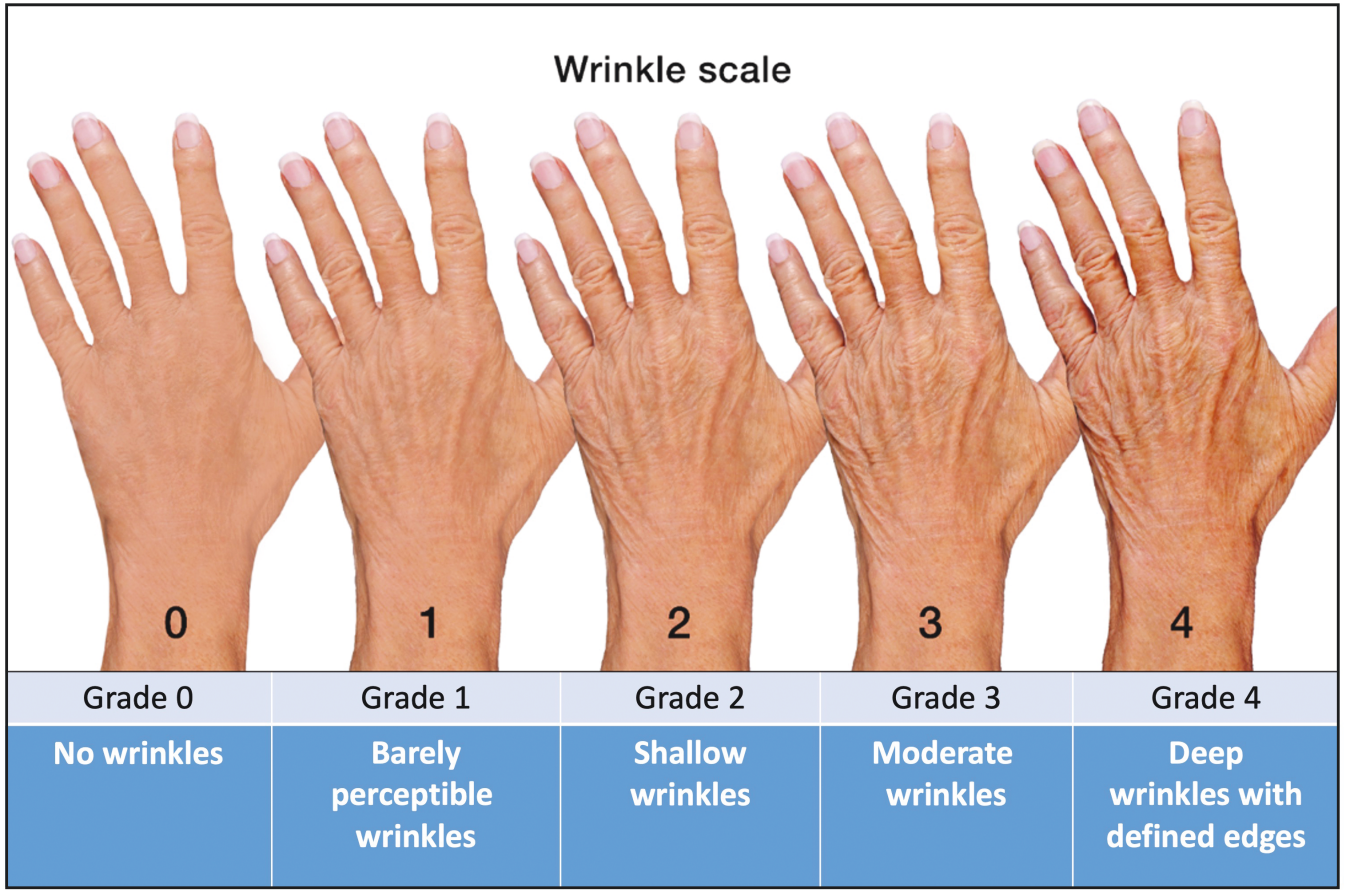
Dorsal hand wrinkle scale.

The Dorsal Hand Wrinkling Scale (Figure 1) is a 5-point photonumeric rating scale. The scale ratings are 0 (no wrinkles), 1 (barely perceptible wrinkles), 2 (shallow wrinkles), 3 (moderate wrinkles), and 4 (deep wrinkles with defined edges). To create the scale images, the left hand of a 64-year-old female, whose photographs were not used for the scale validation portion of this study, with a wrinkle grade of 2, as decided by all members of the study team, was selected. Using Adobe Photoshop (Adobe Systems, San Jose, CA), this base image was morphed to increase or decrease wrinkle severity, creating the remaining 4 images for this scale. All members of the study team agreed that these images aligned with the descriptors for each grade.

**Figure 2.**
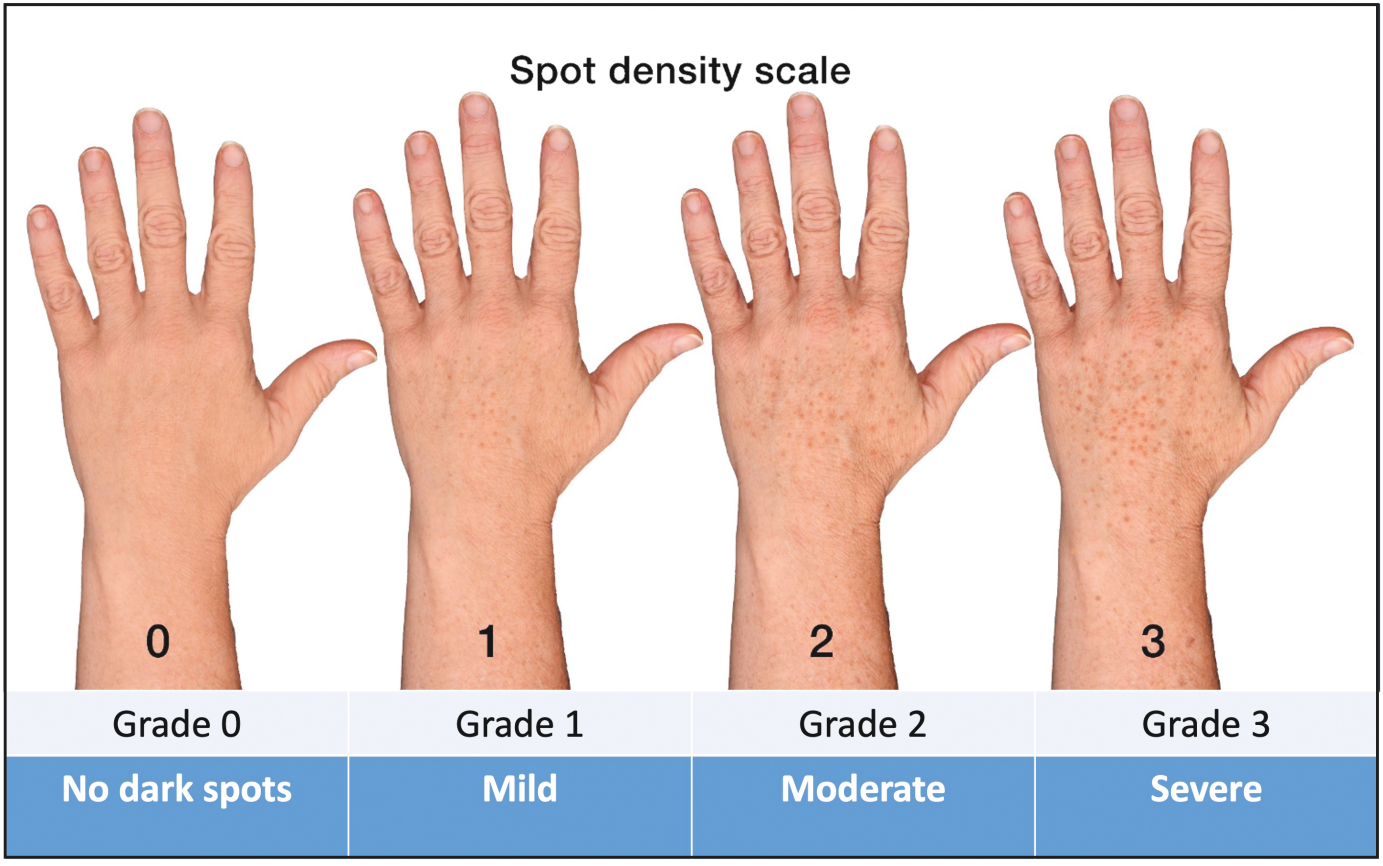
Dorsal hand pigmentation density scale.

**Figure 3.**
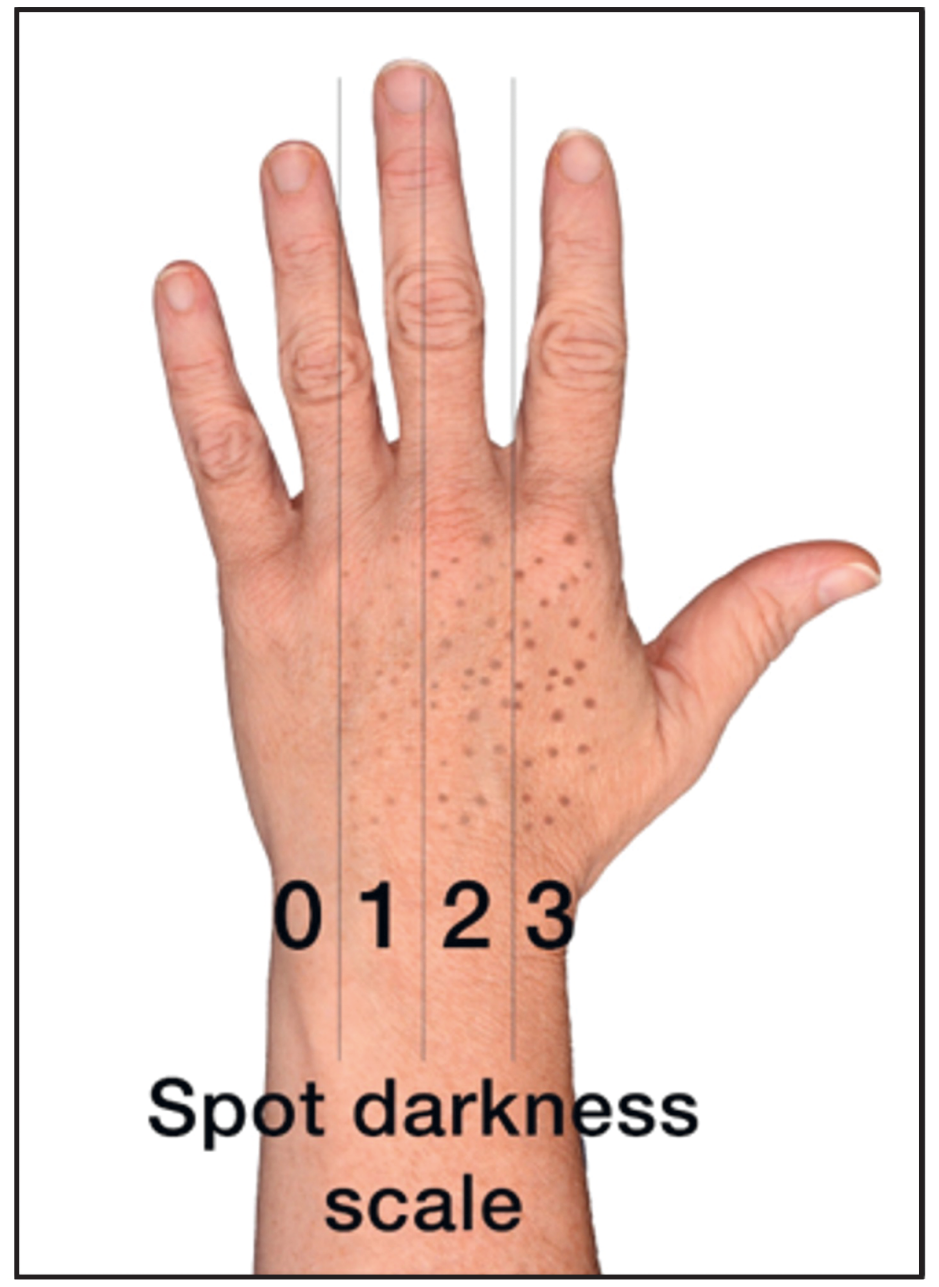
Dorsal hand pigmentation intensity scale.

The Dorsal Hand Pigmentation Density Scale (Figure 2) and the Dorsal Hand Pigmentation Intensity Scale (Figure 3) are 4-point photonumeric scales developed to assess the quantity and darkness of dark spots on the dorsal hand, respectively. The scale ratings are 0 (no dark spots), 1 (mild), 2 (moderate), and 3 (severe). To create the scale images, the left hand of a 61-year-old male, whose photographs were not used for the scale validation portion of this study, with a grade of 1 for both scales, as decided by all members of the study team, was selected. Using Adobe Photoshop, this base image was morphed to change dark spot density and intensity, creating the remaining 3 images for each of these scales. All members of the study team agreed that the features on these images increased in severity with each grade and the image for each scale was easily distinguishable from images for any other grade.

### Evaluator Training and Scale Validation

**Figure 4.**
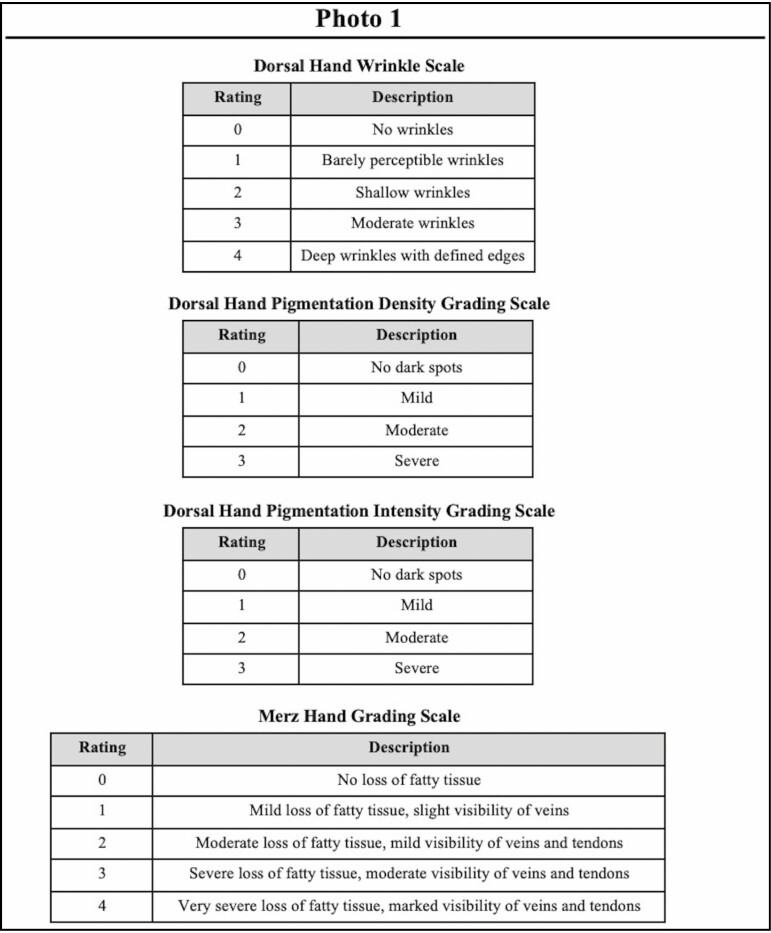
Photo evaluation case report form.

The order of the 100 dorsal hand photographs (50 left hands and 50 right hands) was randomized using www.randomizer.org and arranged on 100 PowerPoint (Microsoft, Redmond, WA) slides. A total of 100 case report forms (CRFs), one for each photograph, were created containing each of the 4 scales (Figure 4). The CRFs were arranged in a binder that was distributed on the day of training, and raters were instructed to not view the binder or PowerPoint before the training session.

**Figure 5.**
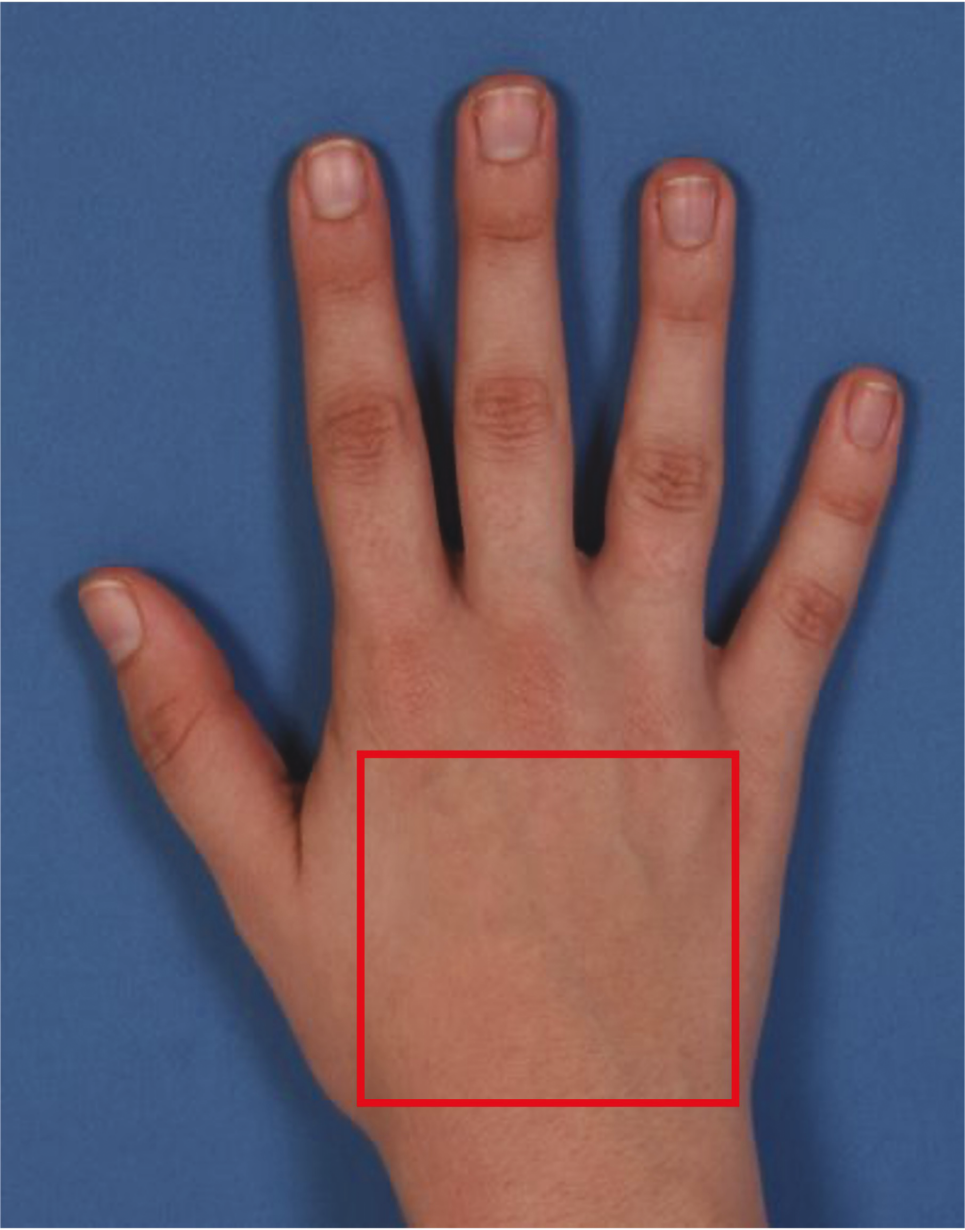
Evaluated area of the dorsal hand.

Eleven physicians in the Department of Plastic Surgery (3 chief residents, 6 senior residents, and 2 aesthetic surgery fellows) were selected to complete the photograph evaluations. Initial training for the first 5 raters was conducted over Zoom (San Jose, CA), as this study was launched during the COVID-19 pandemic and gatherings of more than 5 people were not permissible. In order to increase the power of this study, a second training was provided at a later date for an additional 6 raters, this time in person, utilizing the same training methodology. Before scale validation, all 11 physician raters were trained on the 3 scales under investigation and on the already-validated MHGS in an interactive group training session as well as instructed to evaluate the dorsal hand from the wrist to the metacarpophalangeal joint (Figure 5). The raters scored the 25 photographs using each of the 4 scales and shared their grades with the group. If there were any discrepancies in scoring, a discussion was had until everyone could reach a consensus on a single grade. The results of this training were not included in the statistical analysis.

The 11 raters were then instructed to grade the remaining 75 photographs using all 4 scales. Evaluations were completed independently, with each rater using the same computer screen as the training. Each rater completed a total of 100 CRFs containing a grade for each of the 4 scales.

### Statistical Analysis

Inter-rater variability between paired raters was analyzed using linear weight Kappa scores. Kappa scores are a quantitative measure of agreement between 2+ independent observers evaluating the same item on a scale from 0 to 1. Kappa scores between 0.0 and 0.2 indicate slight agreement, 0.21 and 0.40 fair agreement, 0.41 and 0.60 moderate agreement, 0.61 and 0.80 substantial agreement, and 0.81 and 1.00 almost perfect agreement between raters.^6,15^ Additionally, Kappa scores using Fleiss-Cohen weights were calculated to determine the agreeability of the combined raters. A *P*-value less than 0.05 indicated statistical significance.

## RESULTS

**Figure 6.**
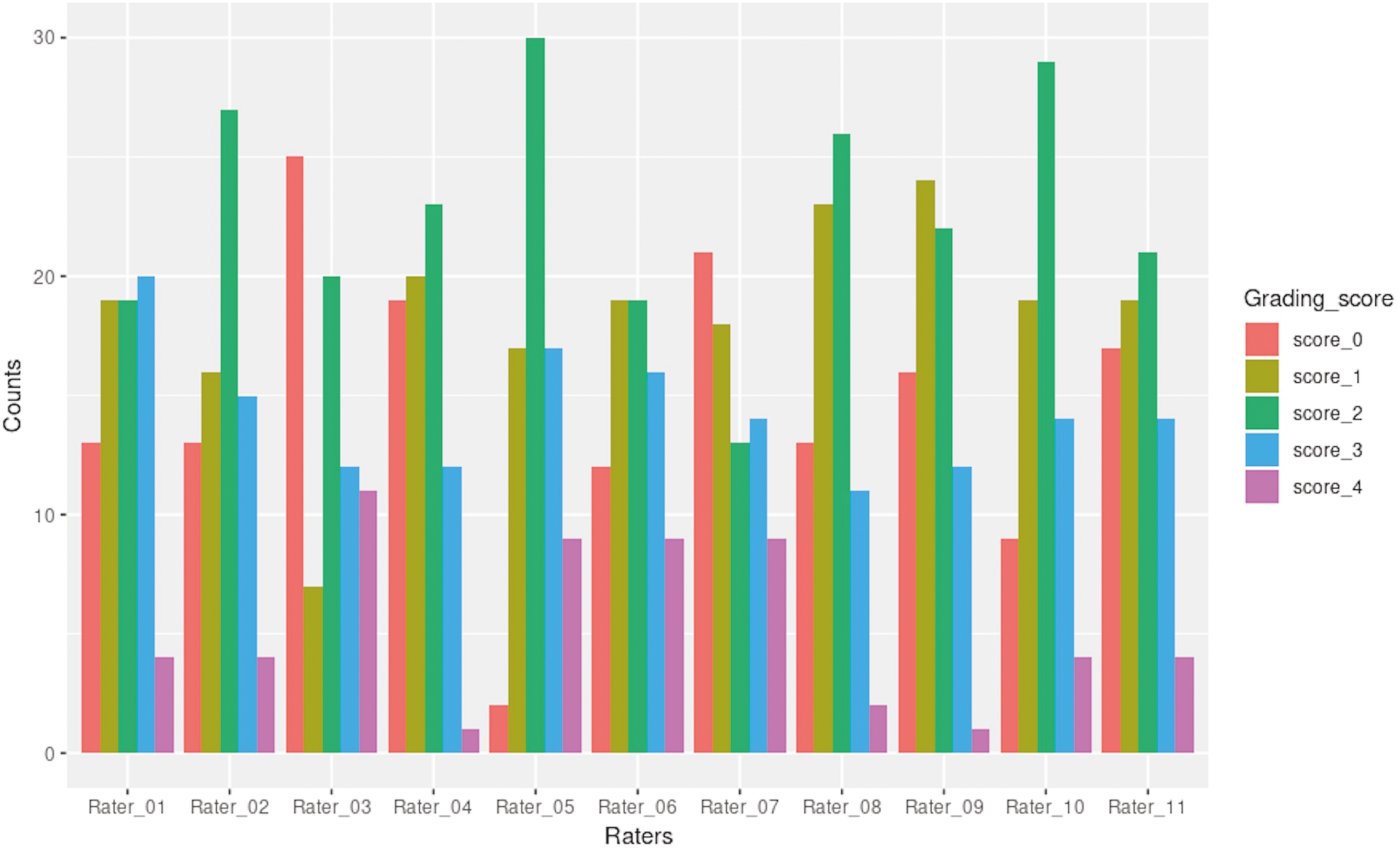
Distribution of grading scores for the dorsal hand wrinkle grading scale.

**Figure 7.**
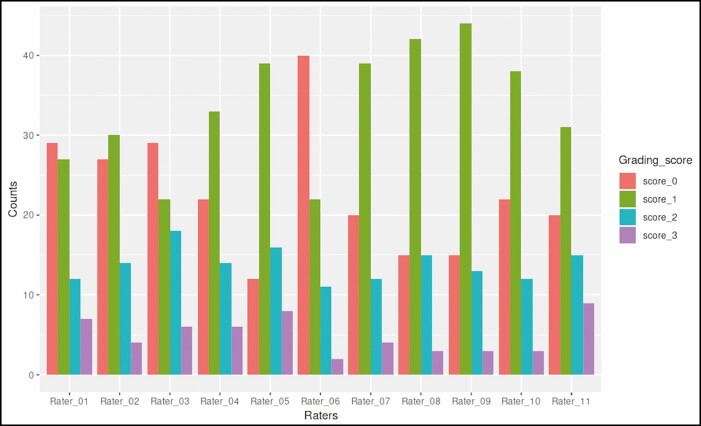
Distribution of grading scores for the dorsal hand pigmentation density grading scale.

**Figure 8.**
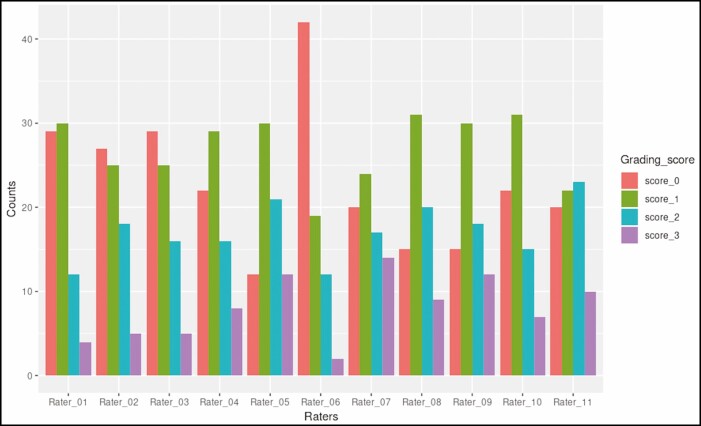
Distribution of grading scores for the dorsal hand pigmentation intensity grading scale.

**Figure 9.**
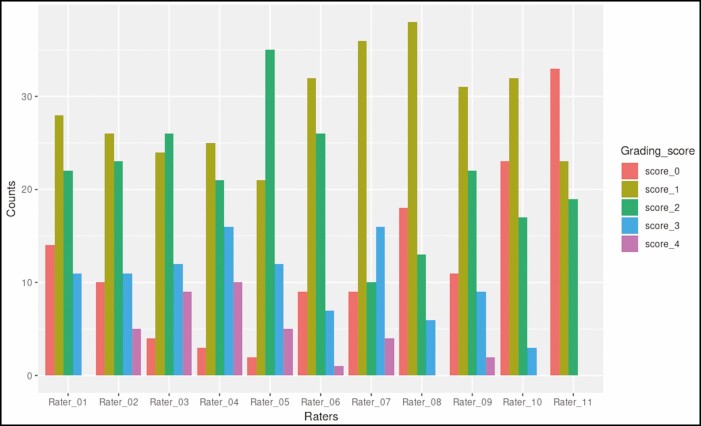
Distribution of grading scores for the merz hand grading scale.

Figures 6-9 illustrate the distribution of grading scores of each rater for each of the 4 scales evaluated. In other words, the count indicates the number of times a rater assigned a specific grade for the scale in question.

**Figure 10.**
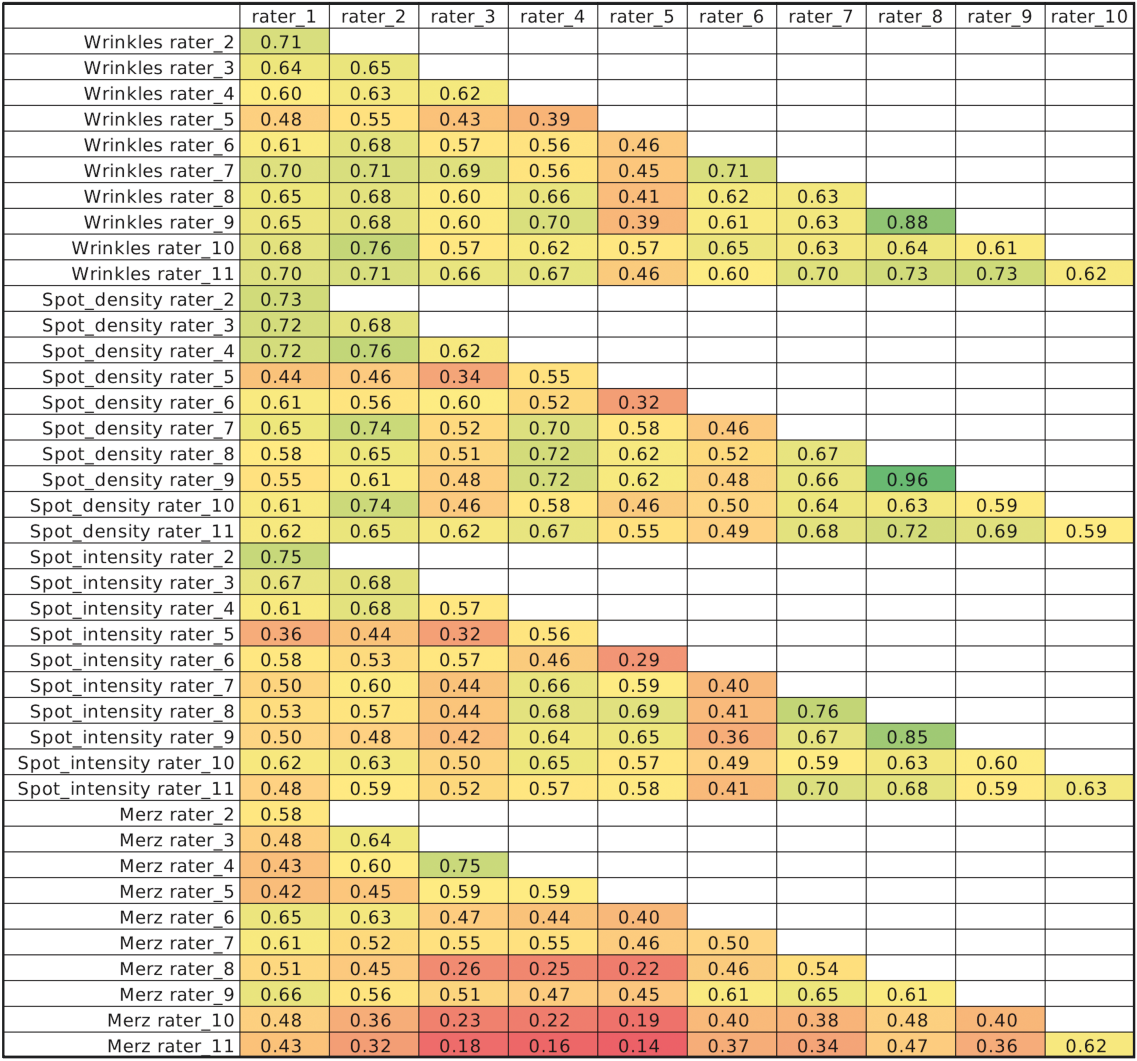
Paired kappa scores.

Kappa scores comparing the scores of 2 raters between each other for the 3 scales under investigation and already-validated MHGS are illustrated in Figure 10. When evaluating all 11 raters, the Kappa score for the MHGS was 0.25, indicating fair agreement; 0.40 for wrinkle scale, indicating fair agreement; and 0.48 and 0.46 for the pigmentation density and intensity scales, respectively, indicating moderate agreement. The *P*-value for all tests was < 0.001.

## DISCUSSION

**Figure 11.**
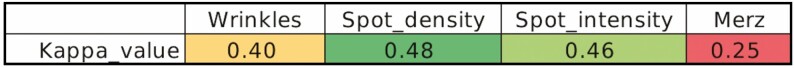
Kappa scores of combined raters using fleiss-cohen weights.

The 3 scales under investigation can be used to reliably and consistently characterize wrinkles and dark spots on the dorsal hand. Combined weighted Kappa scores, a quantitative indicator of inter-rater agreement, were highest for the 2 scales evaluating dorsal hand pigmentation: 0.48 for the Dorsal Hand Pigmentation Density Grading Scale and 0.46 for the Dorsal Hand Pigmentation Density Grading Scale. Furthermore, inter-rater agreement was the lowest for the MHGS, 0.25, indicating only fair agreement (Figure 11).

Upon reviewing the literature, 3 validated scales were found that quantified the severity of hand aging. The 5-point MHGS measures loss of fatty tissue and visibility of tendons and veins. While very few details were given on how the scale was created, the authors proposed that such a tool could provide a standard, objective evaluation of clinical trial outcomes specific to the dorsal hand.^1,3^ Accordingly, this photonumeric scale was used in the Food and Drug Administration (FDA) trial evaluating the correction of dorsal hand volume loss with calcium hydroxyapatite.^1^ The scale was first validated using 35 photographs in 2008,^3^ and in 2015, the study team validated the MHGS for the live assessment using 84 patients. Several members of this study team were later involved in creating the 5-point photonumeric Allergan Hand Volume Deficit Scale, which only assesses the degree of tendon and vein visibility. Instead of one hand image per grade, 3 real- and morphed-patient images over a range of Fitzpatrick skin types were included for each grade. The inter-rater agreement between 8 physicians during a live-patient assessment was high and comparable to results seen in both the photograph validation and live validation studies for the MHGS.^6^ Finally, Lee et al developed the Hand Volume Rating Scale (HVRS) in 2019.^9^ The authors argued that in order to evaluate the outcomes of hand rejuvenation procedures, both hand volume and textural changes must be considered. Therefore, their 5-point photonumeric scale also accounts for the degree of skin roughness.

The 3 scales under investigation in this study expand upon those previously published in the literature. While volume loss is an important consideration, other factors, such as loss of dermal collagen and photoaging, result in other characteristic signs of aging. Several hand rejuvenation therapies, such as laser and light-based procedures, target these concerns. For example, our study team is currently conducting a study investigating the use of intense pulsed light for the treatment of benign pigmented lesions on the dorsal hand (unpublished data). None of the previously published scales would be valuable for assessing the outcome of an Intense Pulsed Light (IPL) series, as the treatment is focused on photoaging and has a negligible effect on volume. This example highlights the need for additional scales that can be used in combination with previously validated scales to provide a well-rounded analysis of aesthetic treatments.

The results of our study show that these 3 new scales are, at a minimum, as reliable as the already-validated MHGS and assess visible signs of dorsal hand aging that could not be previously classified. As the scales currently found in the literature fail to adequately evaluate other factors contributing to the appearance of the aging hand, particularly relating to dyschromia and wrinkling, it is the authors’ hope that these new scales can be used in combination with preexisting validated scales to evaluate several different components of dorsal hand aging more precisely.

Limitations of this study include a patient population with Fitzpatrick skin types I-IV. The authors agreed that pigmentation would be more difficult, if not impossible, to assess on dorsal hands with darker skin tones. Therefore, it was decided that the evaluation would be limited to Fitzpatrick skin types I-IV. Furthermore, the verbal descriptors used for all of the scales are somewhat vague, and evaluation itself is largely subjective in nature, relying on raters’ perceived scoring of photographs. The scales under investigation were developed with both real and morphed patient photographs for each grade. The study team felt that these example photographs were significantly different enough between each grade to provide raters with an adequate guide. Finally, the degree of overlap between several scales cannot be ignored. Raters noted that it was sometimes difficult to differentiate between wrinkles and visible tendons in patient photographs. While the MHGS takes into account the appearance of overall volume depletion in the hand, paying particular attention to volume loss and tendon visibility, it does not independently evaluate the wrinkling of the hand. As these conditions are a result of 2 different processes, loss of dermal collagen vs atrophy of subcutaneous tissue, the study team felt that it is crucial that 2 classification systems exist in an attempt to characterize wrinkles and volume loss as separate entities. An integrated scale including all of these measurements may be useful in creating a single, cohesive assessment; however, the authors agreed that it was important to first create individual scales assessing each component of aging independently. Validation of a single scale including all characteristics may be considered in the future.

Further studies may repeat the analysis with more detailed descriptions for each of the scales. Additionally, intra-rater agreement, where the scales are reevaluated by the same reviewers at a later point in time, would be a valuable addition to the literature. For the sake of conciseness, the intent of this paper was to create the scales and compare them first to an already-validated scale. Intra-rater evaluation may be performed in a future study.

## CONCLUSIONS

The results of this study demonstrate the reliability of 3 new scales that characterize the appearance of wrinkles and dark spots on the dorsal hand. Interobserver agreement was fair to moderate for each of the 3 scales under investigation and similar to that seen with the MHGS assessment. Clinical evaluations of the aging hand, therefore, no longer have to be limited to assessing the volume loss. These 3 scales provide simple tools that can be used in combination with the preexisting validated scales to more accurately classify the aging hand as well as evaluate multiple changes associated with aesthetic hand treatments.
